# Clinical and psychometric characteristics of depression in the elderly

**DOI:** 10.1192/j.eurpsy.2024.1125

**Published:** 2024-08-27

**Authors:** R. Albu-Kalinovic, M.-A. Pop, N.-S. Oana, B. Adela, V. Gabriela-Ionela, E. Virgil-Radu

**Affiliations:** ^1^ UMFVBT; ^2^SCJUPBT, Timisoara, Romania

## Abstract

**Introduction:**

Depression in the elderly represents a multifaceted and critical area of study within the realm of geriatric mental health. As the global population continues to age, the prevalence and impact of depression among older adults have garnered increased attention from researchers and clinicians.

**Objectives:**

This abstract delves into the comprehensive exploration of the clinical and psychometric characteristics of depression in the elderly population. This study aims to contribute to a deeper understanding of depression’s manifestation in the third age, providing invaluable insights that can inform tailored interventions, improve diagnostic accuracy, and enhance the overall quality of life for older adults.

**Methods:**

A cross-sectional study was conducted that gathered 80 patients. Their common characteristics were the signing of the consent, their admission to the Psychiatry Clinic in Timișoara and their main diagnosis with one of the ICD-10 codes F32.x, F33.x or F06.8.

This selection resulted in three groups: patients younger than 65 years old, patients older than 65 years in whom depression began before this age, and patients older than 65 years in whom depression began after 65 years of age. Anamnestic data, paraclinical, socio-demographic data, psychometric scales that measured the level of depression as well as personality scales were collected. The data that was obtained was compared and examined to find significant correlations between the 3 batches.

**Results:**

The results show that there are no significant differences between patients with depression from rural or urban areas, showing the universality of the occurrence of this disorder among the population, regardless their environment. Depression in the third age is most often found in the elderly who have only finished secondary school, education levels playing a role in depression prevalence suggest the significance of socio-economic factors, warranting targeted outreach and education efforts in vulnerable populations. Correlations were also found between the level of depression and certain blood parameters. The integration of these findings for an understanding of the etiology of depression can pave the way for new therapeutic approaches. Certain personality traits were correlated more with certain items on the scales that evaluated depression, thus in those with a neurotic personality it is very strongly correlated with the appearance of guilt as a symptom, agreeableness with psychomotor slowness, paranoid with insomnia, and anankastic and dependent personalities were correlated much more with social isolation.

**Conclusions:**

By recognizing the distinct clinical features and implications of depression in older adults, we can pave the way for improved mental health care and better quality of life for this growing population. This study reinforces the importance of continuous research and collaboration in the field of geriatric mental health.

**Disclosure of Interest:**

None Declared

